# Adalimumab regulates intracellular TNFα production in patients with rheumatoid arthritis

**DOI:** 10.1186/ar4615

**Published:** 2014-07-18

**Authors:** Carlos Zamora-Atenza, Cesar Diaz-Torne, Carme Geli, Cesar Diaz-Lopez, M Angels Ortiz, Patricia Moya, Ivan Castellví, Juan C Nieto, Elisabet Cantó, Jordi Casademont, Candido Juarez, Josep M Llobet, Silvia Vidal

**Affiliations:** 1 Department of Immunology, Biomedical Research Institut Sant Pau (IBB Sant Pau), C. Antoni M. Claret, 167, 08025 Barcelona, Spain; 2 Unit of Rheumatology, Department of Internal Medicine, Hospital de la Santa Creu i Sant Pau, C. Antoni M. Claret, 167, 08025 Barcelona, Spain; 3 Department of Immunology, Hospital de la Santa Creu i Sant Pau, C. Antoni M. Claret, 167, 08025 Barcelona, Spain

## Abstract

**Introduction:**

Adalimumab is a fully human anti–tumor necrosis factor α (anti-TNFα) monoclonal antibody that specifically blocks the interaction of TNFα with its receptors. It binds both soluble and transmembrane TNFα. We hypothesized that blocking these TNFα signals regulates the altered TNFα production in rheumatoid arthritis (RA) patients.

**Methods:**

We compared, by flow cytometry, Toll-like receptor induction levels of membrane and intracellular TNFα in monocytes (iTNFα + CD14+ cells) from 12 patients before and after adalimumab treatment with those from 5 healthy donors.

**Results:**

Before starting the treatment, the percentage of iTNFα+ CD14+ cells in the RA patients was significantly lower than that in healthy donors (mean ± SEM = 33.16 ± 4.82% vs 66.51 ± 2.4%, *P* < 0.001). When we added *in vitro* TNFα to healthy donor culture cells, levels of iTNFα+ CD14+ cells decreased, suggesting that the TNFα signal was responsible for the iTNFα+ CD14+ cell downregulation observed in the RA patients. After 2, 6 and 12 adalimumab injections, we observed significant blocking of membrane and soluble TNFα and a progressive increase in iTNFα+ CD14+ cells in ten patients with a good to moderate response as defined by the European League Against Rheumatism (EULAR) criteria. Levels of iTNFα+ CD14+ cells after 12 injections in these 10 patients were comparable to levels in healthy donors. In two patients, iTNFα+ CD14+ cell upregulation was not observed, and their EULAR-defined responses had not improved. The first patient developed antiadalimumab antibodies, explaining why adalimumab was not able to block membrane and soluble TNFα. In the second patient, adalimumab was discontinued because of adverse effects, which led to a decrease in iTNFα+ CD14+ cells to levels measured before treatment.

**Conclusions:**

Our findings suggest that adalimumab treatment in RA patients can return iTNFα levels to those of healthy donors. This effect was not observed in the presence of neutralizing antiadalimumab antibodies.

## Introduction

Tumor necrosis factor α (TNFα) is a proinflammatory cytokine produced mainly by activated monocytes, macrophages, T lymphocytes and natural killer (NK) cells. In target cells, this cytokine plays a key role in apoptosis, cell survival, immunity and inflammation [1-3]. TNFα is initially synthesized and expressed as a transmembrane protein. Its extracellular proportion is released in the form of a soluble 17 kDa molecule when it is cleaved by a metalloproteinase TNFα-converting enzyme (TACE) [4].

The level of TNFα in the synovial fluid in rheumatoid arthritis (RA) is high [5,6]. In the synovia, TNFα contributes to joint destruction by attracting leukocytes, inducing inflammatory cytokines, upregulating adhesion molecules on endothelial cells and activating the synthesis of metalloproteinases in synovial macrophages, fibroblasts and chondrocytes [7-10]. TNFα also plays a role in osteoclastic bone resorption. It stimulates osteoclastogenesis by differentiating progenitor cells and enhancing the expression of receptor activator of nuclear factor κB ligand [11].

In view of these findings, it is not surprising that TNFα has become a strategic target in the treatment of RA patients. Adalimumab is a fully human neutralizing anti-TNFα monoclonal antibody that specifically blocks the interaction of TNFα with p55 and p75 cell-surface TNFα receptors [12]. By blocking TNFα, adalimumab can attenuate cartilage and bone destruction partially through the downregulation of matrix metalloproteinases [13]. Moreover, adalimumab can reduce acute-phase reactants of inflammation [14], inflammatory cytokines [10] and adhesion molecules responsible for leukocyte migration [13]. Studies have shown that adalimumab is effective in preventing joint damage in early RA [15] and improving clinical and laboratory parameters, emphasizing the pivotal role of TNFα in this pathology. However, not all RA patients treated with adalimumab show this clinical response [16].

Although adalimumab has been found to block soluble and transmembrane TNFα [17], whether adalimumab can regulate the production of intracellular TNFα (intracellular TNFα) on monocytes is unknown. Our aim in this study was to determine whether blocking TNFα signals regulates the TNFα production in patients with RA.

## Methods

### Samples

Heparinized blood obtained from healthy donors (HDs) (*n* = 5), patients with active RA who had never received biological therapy (*n* = 12), RA patients in remission or with low activity who were being treated with methotrexate (*n* = 3) and similar patients treated with infliximab (*n* = 3) was collected in BD Vacutainer tubes (BD Pharmingen, Franklin Lakes, NJ, USA). Diagnosis of RA was based on the American College of Rheumatology criteria [18]. Disease activity was measured using the Disease Activity Score in 28 joints erythrocyte sedimentation rate (DAS28-ESR) [19]. Table 1 shows demographic data, clinical parameters and laboratory values of patients with active RA who were receiving adalimumab treatment every 2 weeks. We collected blood from RA patients after two (4 weeks), six (12 weeks) and twelve (24 weeks) injections of adalimumab. To determine the effectiveness of adalimumab treatment, we evaluated clinical improvement using the European League Against Rheumatism (EULAR) response criteria [20]. We also collected blood from three methotrexate-treated RA patients over the course of more than 3 years (ESR (mean ± SD) = 36.33 ± 16.65 mm/h, C-reactive protein (CRP) = 18.13 ± 15.15 mg/L) and three RA patients who had been treated with infliximab for more than 5 years (ESR = 42 ± 30.34 mm/h, CRP = 9.17 ± 3.32 mg/L). Written informed consent was obtained from the participants, and ethical approval of the study was granted by the institutional ethics committee of the Hospital de la Santa Creu i Sant Pau.

**Table 1:** **Baseline characteristics of rheumatoid arthritis patients treated with adalimumab**^**a**^

**Characteristics**	**Data**
Gender (% women)	92.8
Mean age (±SD), yr	56.6 ± 13.6
Mean disease duration (±SD), yr	13.8 ± 11.2
Mean RF + and/or ACPA + (%)	78.6
Mean concomitant methotrexate (%)	64.3
Mean concomitant prednisone (%)	64.3
Mean previous DMARDs, number (range)	3.07 (1 to 5)
Mean ESR, mm/h (±SD)	40.1 ± 23.6
Mean CRP, mg/L (±SD)	15.6 ± 17.8
Mean DAS28-ESR, IU (±SD)	5.05 ± 0.95

^a^ACPA Anticitrullinated peptide antibodies; CRP, C-reactive protein; DAS28-ESR, Disease Activity Score in 28 joints erythrocyte sedimentation rate; DMARDs, Disease-modifying arthritis drugs; RF, Rheumatoid factor.

### Determination of intracellular tumor necrosis factor α production in leukocytes

Whole blood from HDs and RA patients was cultured in 5-ml polypropylene tubes (BD Biosciences, San Jose, CA, USA) with RPMI 1640 medium, 1 μg/ml lipopolysaccharide (LPS) or 10 μg/ml lipoteichoic acid (LTA) (InvivoGen, San Diego, CA, USA) in the presence of BD GolgiStop™ Protein Transport Inhibitor (BD Biosciences) for 4 hours. After culturing, cells were stained with anti-CD14-fluorescein isothiocyanate (anti-CD14-FITC) (ImmunoTools, Friesoythe, Germany). Samples were fixed and lysed with FACS Lysing Solution (BD Biosciences) for 10 minutes. Samples were then permeabilized with phosphate-buffered saline plus 0.3% saponin. After permeabilization, samples were washed and stained with anti-TNFα-phycoerythrin (anti-TNFα-PE) (BioLegend, San Diego, CA, USA). iTNFα production was analyzed in CD14+ cells and in CD14- neutrophils and lymphocytes by flow cytometry.

### Determination of membrane tumor necrosis factor α on monocytes

Whole blood of HDs and RA patients was cultured in RPMI 1640 medium, LPS (1 μg/ml) or LTA (10 μg/ml) in the presence of 25 μl of metalloprotease TNFα–converting enzyme (TACE) inhibitor (Cytognos, Salamanca, Spain). After 4 hours in culture, cells were stained with anti-CD14-FITC and anti-TNFα-PE (BioLegend). Samples were then incubated with 2 ml of QUICKLYSIS™ solution (Cytognos). Membrane TNFα (mTNFα) was analyzed on CD14+ cells by flow cytometry. Adalimumab injections blocked mTNFα, and only free mTNFα was detected. When patient serum (and consequently adalimumab) was washed out of Toll-like receptor (TLR) ligand cultures, mTNFα was detectable (data not shown).

### Determination of tumor necrosis factor α levels in cultures supernatants

Whole blood of HDs and RA patients was cultured with medium, LPS (1 μg/ml) or LTA (10 μg/ml) at 5% CO_2_ and 37°C. After 24 hours in culture, supernatants were collected. Soluble TNFα levels were determined using a specific enzyme-linked immunosorbent assay (ELISA) kit according to the manufacturer’s instructions (BD Biosciences). TNFα was quantified with standard curves, and the limit of detection was 7.5 pg/ml. Adalimumab injections blocked soluble TNFα, and only free soluble TNFα was detected.

### Determination of adalimumab levels and antiadalimumab antibodies in serum

Serum from RA patients was collected and kept at -80°C until use. Adalimumab levels and antiadalimumab antibodies were determined using a specific ELISA kit according to the manufacturer’s instructions (Proteomika, Derio, Spain). The limit of detection was 0.15 ng/ml for adalimumab and 0.4 AU/ml for antiadalimumab antibodies.

### Flow cytometry analysis

iTNFα and mTNFα were analyzed on CD14+ monocytes, and iTNFα was analyzed in neutrophils and lymphocytes gated according to forward-scatter and side-scatter parameters in CD14- cells using a Beckman Coulter FC500 flow cytometer (Beckman Coulter, Barcelona, Spain). We calculated the percentages of positive cells (% cells) and geometric mean fluorescence intensity (GMFI) of each marker using CXP Software 2.2 (Beckman Coulter). The integrated geometric mean fluorescence intensity (iGMFI) was used to determine the amount of mTNFα produced by CD14+ cells and was calculated by multiplying the percentage of positive cytokine-producing cells by the GMFI [21].

### Statistical analysis

Statistical analyses were performed using paired *t*-tests, *t*-tests and Spearman’s correlation in GraphPad Prism 5 software (GraphPad Software, La Jolla, CA, USA). Data are presented as mean and ± SEM. *P*-values <0.05 were considered statistically significant.

## Results

### Tumor necrosis factor α production in rheumatoid arthritis patients

iTNFα and mTNFα levels in CD14+ cells and TNFα levels in supernatants of LPS (TLR4 ligand) and LTA (TLR2 ligand) of HDs were compared with those of RA patients. For this purpose, whole-blood cells obtained from HDs and RA patients were cultured with TLR ligands before adalimumab therapy was started.

**Figure 1:**
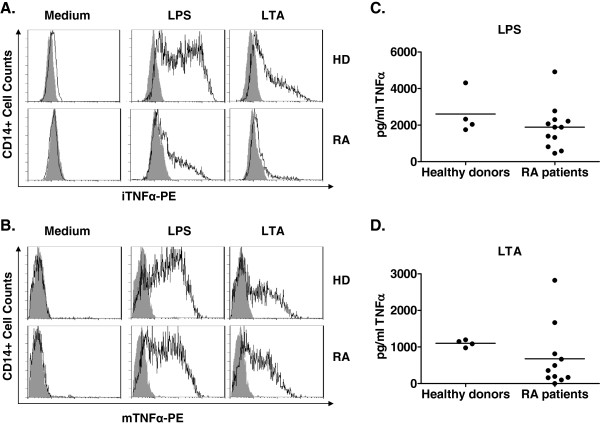
**Production of tumor necrosis factor α in healthy donors and rheumatoid arthritis patients after lipopolysaccharide and lipoteichoic acid cultures.** Whole blood of healthy donors (HDs) (*n* = 4) and rheumatoid arthritis (RA) patients (*n* = 12) collected before adalimumab treatment was cultured in the presence of lipopolysaccharide (LPS; 1 μg/ml) or lipoteichoic acid (LTA; 10 μg/ml) for 4 hours to analyze intracellular tumor necrosis factor α–phycoerythrin (iTNFα-PE) and membrane tumor necrosis factor α–phycoerythrin (mTNFα-PE) by flow cytometry and for 24 hours to analyze levels of TNFα in supernatants by ELISA. **(A)** iTNFα and **(B)** mTNFα were analyzed in monocytes from LPS and LTA cultures gated according to CD14 expression. Levels of TNFα in culture supernatants of LPS **(C)** and LTA **(D)** were analyzed in HDs and RA patients. A *t*-test was used for statistical analysis.

HDs showed a higher percentage of iTNFα+ CD14+ cells than RA patients after LPS or LTA culture (LPS = 66.51 ± 2.4 for HDs vs 33.16 ± 4.82 for RA patients, *P* < 0.001; LTA = 39.92 ± 1.42 for HDs vs 17.9 ± 3.4 for RA patients, *P* < 0.01) (Figure 1A). TLR ligand–stimulated neutrophils from HDs showed a higher percentage of iTNFα+ than those from RA patients (LPS = 0.81 ± 0.15% for HDs vs 0.35 ± 0.05% for RA patients, *P* < 0.01; LTA = 0.55 ± 0.1% for HDs vs 0.21 ± 0.03% for RA patients, *P* < 0.05). HDs and RA patients had a similar percentage of iTNFα+ lymphocytes (LPS = 0.20 ± 0.04% for HDs vs 020 ± 0.04% for RA patients; LTA = 0.10 ± 0.02% for HDs vs 0.12 ± 0.02% for RA patients).The percentages of mTNFα+ CD14+ cells and the levels of supernatant TNFα in RA patients did not differ from those of HDs after TLR ligand cultures (Figure 1B to D).

### Intracellular tumor necrosis factor α production in rheumatoid arthritis patients after adalimumab treatment

**Figure 2:**
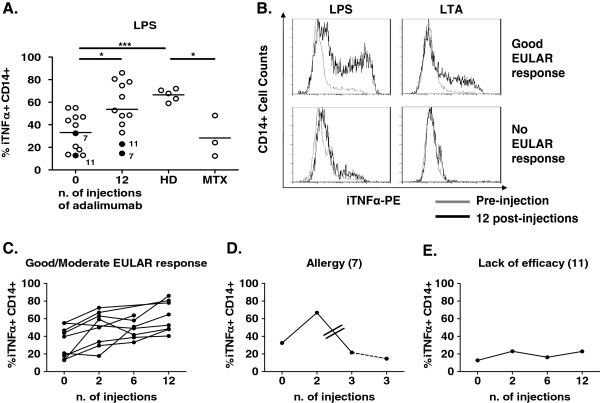
**Intracellular tumor necrosis factor α production in whole-blood cells from healthy donors and rheumatoid arthritis patients after adalimumab treatment.** Intracellular tumor necrosis factor α (iTNFα) production was analyzed on CD14+ cells in rheumatoid arthritis (RA) patients before treatment and after 2, 6 and 12 injections of adalimumab. **(A)** This graph shows the percentage of iTNFα+ CD14+ after lipopolysaccharide (LPS) culture in patients before and after adalimumab treatment, after methotrexate (MTX) treatment and in healthy donors (HDs). **(B)** This representative image of flow cytometry results shows the percentage of iTNFα+ CD14+ cells after LPS and lipoteichoic acid (LTA) culture in RA patients before and after 12 injections of adalimumab. EULAR, European League Against Rheumatism. **(C)** This graph shows the percentage of iTNFα+ CD14+ cells in LPS culture before and after 2, 6 and 12 injections of adalimumab into RA patients with a good to moderate EULAR-defined response. **(D)** This graph shows the percentage of iTNFα+ CD14+ cells in LPS culture before and after two and three injections of adalimumab into patient 7. The two lines in this figure correspond to two missed injections of adalimumab. The dashed line corresponds to the suppression of adalimumab. **(E)** This graph shows percentage of iTNFα+ CD14+ cells in LPS culture before and after 2, 6 and 12 injections of adalimumab into patient 11. A paired *t*-test was used for statistical analysis between RA patients at different time points. A *t*-test was used for statistical analysis between RA patients and HDs. *P < 0.05, ***P < 0.001.

We next sought to determine how adalimumab treatment could modify the percentage of iTNFα in CD14+ monocytes of RA patients. Whole blood from RA patients collected after 2, 6 and 12 injections of adalimumab was cultured with TLR ligands, and iTNFα+ CD14+ cell percentages were determined. Twelve injections of adalimumab significantly increased the iTNFα+ CD14+ percentages in RA patients up to levels of HDs (33.16 ± 4.82% for preinjection vs 53.69 ± 23.49% after 12 injections, *P* < 0.05) (Figure 2A). Levels of iTNFα+ CD14+ cells increased after two (48.29 ± 20.45%, *P* < 0.01) and six injections (42.01 ± 18.93%, *P* < 0.05). The kinetics were similar in LTA cultures (17.91 ± 11.78% for preinjection vs 32.51 ± 21.46% after 12 injections, *P* < 0.05). A higher percentage of iTNFα+ neutrophils was observed after 12 infusions of adalimumab (LPS = 0.74 ± 0.13%, *P* < 0.05; LTA = 0.47 ± 0.09%, *P* < 0.05).

To confirm the adalimumab findings with other comparable anti-TNFα treatments, we included RA patients treated with infliximab. The percentages of iTNFα+ CD14+ cells were comparable in infliximab-treated patients (71.53 ± 1.53%), in patients treated with adalimumab (60.68 ± 5.87%) and in HDs (66.51 ± 2.4%).

To determine whether the increases in iTNFα+ CD14+ cells observed in the RA patients were due to adalimumab treatment or a consequence of the remission state, we analyzed RA patients in methotrexate-induced remission. Percentages of iTNFα+ CD14+ cells in RA patients in methotrexate-induced remission were significantly lower than those in RA patients after 12 injections of adalimumab treatment (28.25 ± 10.5% and 60.68 ± 5.87%, respectively, *P* < 0.05) (Figure 2A). These low percentages of iTNFα+ CD14+ cells in methotrexate-treated patients were not restored by overnight culture with adalimumab (23.89 ± 13.94%).

### Intracellular tumor necrosis factor α regulation in rheumatoid arthritis patients in good to moderate responders to adalimumab

After 12 injections of adalimumab, ESR, CRP and DAS28-ESR were significantly lower than before the injections (respectively, 21.72 ± 14.83 mm/h, *P* < 0.05; 2.64 ± 2.22 mg/L, *P* < 0.05; and 2.89 ± 0.96 UI, *P* < 0.001). The percentage of iTNFα+ CD14+ cells was comparable in HDs and RA patients with a good to moderate EULAR response (10 of 12 patients) after 12 injections of adalimumab (LPS = 60.68 ± 5.87% vs 66.51 ± 2.4%). These patients had already presented increased percentages of iTNFα+ CD14+ cells after two (49.13 ± 7.05%) and six injections (47.91 ± 4.09%) (Figure 2C).

Adalimumab was not effective in two patients. Patient 11 presented a lower percentage of iTNFα+ CD14+ cells after LPS and LTA cultures (data not shown) at all postinjection time points than HDs (Figure 2E). Another patient, patient 7, received the third injection after a 4-week delay due to an infection. The patient then presented adverse effects (allergy), and adalimumab was discontinued. In this patient, the increased percentage of iTNFα+ CD14+ cells and higher EULAR-defined response seen after the first two injections were not observed after the third injection (Figure 2D). Consistent with our observations, DAS28 levels and ESR were inversely correlated with the percentage of iTNFα+ CD14+ cells along treatment (DAS28-ESR = *R* = -0.445, *P* = 0.0095; ESR = R = -0.563, *P* < 0.0001) in good to moderate EULAR-defined response patients.

### Adalimumab levels and antiadalimumab antibodies in serum of rheumatoid arthritis patients

In view of the findings described above, we next compared adalimumab levels and antiadalimumab antibodies in patients 7 and 11 with those in RA patients with good to moderate EULAR-defined responses. The seven patients with good to moderate EULAR-defined responses presented therapeutic levels of adalimumab (>1 μg/ml) [22] after 12 injections and did not have detectable antiadalimumab antibody levels in serum (Table 2).

**Table 2:** **Adalimumab and antiadalimumab antibody levels in serum of rheumatoid arthritis patients**^**a**^

**Patient**	**Adalimumab levels (mg/ml)**			**Antiadalimumab levels (AU/ml)**			**Discontinuation cause**
	**4 wk**	**12 wk**	**24 wk**	**4 wk**	**12 wk**	**24 wk**	
1	n.d.	n.d.	n.d.	n.d.	n.d.	n.d.	–
2	n.d.	n.d.	n.d.	n.d.	n.d.	n.d.	–
3	n.d.	n.d.	9.62	n.d.	n.d.	NG	–
4	n.d.	n.d.	10.06	n.d.	n.d.	NG	–
5	n.d.	n.d.	4.9	n.d.	n.d.	NG	–
6	n.d.	n.d.	14.697	n.d.	n.d.	NG	–
8	14	14.4	24.065	NG	NG	NG	–
9	n.d.	n.d.	39.409	n.d.	n.d.	NG	–
10	n.d.	n.d.	n.d.	n.d.	n.d.	n.d.	–
12	n.d.	n.d.	8	n.d.	n.d.	NG	–
7	7.8	7	NG	NG	NG	NG	Allergy
11	NG	NG	NG	297,268	81,833	2,925	Lack of efficacy

^a^n.d., Not determined; NG, Negative.

The two patients (patients 7 and 11) who did not show a EULAR-defined response presented different patterns. Although patient 7 showed therapeutic levels of adalimumab after two and three injections, therapy was suspended after the third injection due to allergic reactions. Consequently, at 24 weeks after initiation of treatment (14 weeks from the last adalimumab injection), adalimumab levels were undetectable, coinciding with the downregulation of iTNFα+ CD14+ percentages to levels comparable to those before treatment. Patient 11 presented no clinical response to adalimumab at any moment during follow-up, coinciding with undetectable levels of adalimumab in serum after 2, 6 and 12 injections. These undetectable levels of adalimumab were a consequence of the high titers of antiadalimumab antibodies after the second injection (Table 2). No patients had detectable levels of adalimumab antibodies in serum before adalimumab treatment.

### Free tumor necrosis factor α levels at the membrane of monocytes and in the culture supernatants from rheumatoid arthritis patients

**Figure 3:**
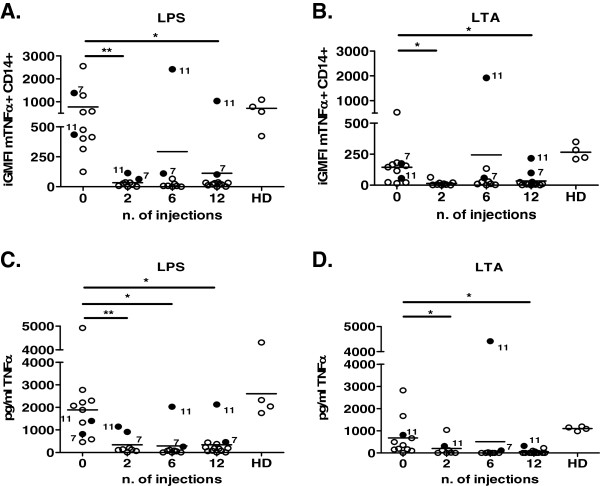
**Free tumor necrosis factor α levels on membranes of monocytes and culture supernatants from healthy donors and rheumatoid arthritis patients after adalimumab treatment.** Membrane tumor necrosis factor α (mTNFα) levels on monocytes and culture supernatants were analyzed in rheumatoid arthritis (RA) patients before and after 2, 6 and 12 injections of adalimumab. Graphs show integrated geometric mean fluorescence intensity (iGMFI) of mTNFα in healthy donors (HDs) and RA patients after lipopolysaccharide (LPS) culture **(A)** and lipoteichoic acid (LTA) culture **(B)**. Graphs shows TNFα concentrations (pg/ml) in healthy donors and RA patients at all time points of adalimumab treatment, after LPS culture **(C)** and LTA culture **(D)**. A paired *t*-test was used for statistical analysis between RA patients and number of injections. A *t*-test was used for statistical analysis between RA patients and healthy donors. *P < 0.05, **P < 0.01.

We evaluated the iGMFI of mTNFα on monocytes and soluble TNFα in TLR ligand culture supernatants after 2, 6 and 12 injections of adalimumab. mTNFα+ CD14+ cell iGMFI decreased after two injections in all patients (LPS = 773.7 ± 212.9 preinjection, 33.51 ± 12.03 for two injections (*P* < 0.01), 293.3 ± 265.8 for six injections, and 112.1 ± 84.39 for twelve injections (*P* < 0.05); LTA = 144.4 ± 43.68 preinjection, 13.79 ± 6.63 for two injections (*P* < 0.05), 243.4 ± 210.1 for six injections and 34.14 ± 18.27 for twelve injections (*P* < 0.05)) (Figure 3A and B). In patient 11, mTNFα+ CD14+ iGMFI levels decreased after two injections, but increased thereafter to the levels measured before initiation of treatment. As expected, iGMFI levels of mTNFα+ on CD14+ cells from RA patients in remission after infliximab were comparable to those from RA patients in remission after adalimumab, but they were significantly different from those of HDs (41.99 ± 16.28 for infliximab, 20.62 ± 4.02 for adalimumab and 715.4 ± 144.6 for HDs; *P* < 0.01).

After two injections of adalimumab, levels of free soluble TNFα significantly decreased in supernatants of TLR cultures in all but patient 11 (LPS = 1,888 ± 378.5 pg/ml preinjection, 338.7 ± 153.6 pg/ml for two injections (*P* < 0.01), 290.5 ± 219.1 pg/ml for six injections (*P* < 0.05) and 338.9 ± 155.4 pg/ml for twelve injections (*P* < 0.05); LTA = 676.3 ± 257.4 pg/ml preinjection, 202.2 ± 125.4 pg/ml for two injections (*P* < 0.05), 506.7 ± 488.6 pg/ml for six injections, and 54.37 ± 28.49 pg/ml for twelve injections (*P* < 0.05)) (Figure 3C and D).

### Regulation of intracellular tumor necrosis factor α production by tumor necrosis factor α signaling

**Figure 4:**
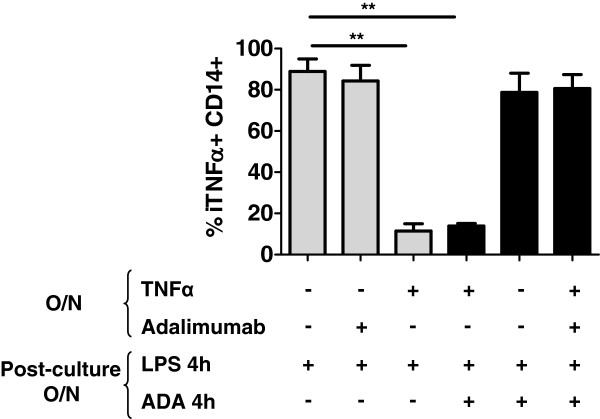
**Intracellular tumor necrosis factor α regulation in monocytes after tumor necrosis factor α culture overnight.** Whole blood of healthy donors was cultured overnight with tumor necrosis factor α (TNFα; 6 ng/ml), adalimumab (ADA; 2 μg/ml) or media. After culturing, whole blood was stimulated with lipopolysaccharide (LPS) or LPS plus adalimumab for 4 hours. The percentage of iTNFα+ CD14+ was analyzed. O/n, Overnight. A *t*-test was used for statistical analysis. **P < 0.01.

We next analyzed how TNFα-TNF receptor signaling downregulated iTNFα production in CD14+ cells. Overnight preincubation of whole-blood cells from HDs with recombinant TNFα (6 ng/ml) decreased the percentage of iTNFα+ CD14+ cells in a 4-hour LPS culture (88.87 ± 6.01% for medium culture vs 11.49+2.11 for TNFα culture; *P* < 0.01).To understand the effect of adalimumab on iTNFα regulation by TNFα signaling, we established two conditions. First, we precultured cells with TNFα plus adalimumab (2 μg/ml) overnight before stimulation with LPS plus adalimumab for 4 hours. Adalimumab restored the production of iTNFα in CD14+ monocytes to normal levels (80.59 ± 6.79%). Second, we precultured cells overnight with TNFα before stimulation with LPS in the presence of adalimumab for 4 hours. In this case, adalimumab was not able to restore iTNFα production in CD14+ cells (13.91 ± 0.18%). Preculturing cells with adalimumab did not alter iTNFα+ CD14+ percentages (Figure 4).

## Discussion

Our present findings suggest that, as a result of exposure to TNFα, cells from patients with active RA had a lower percentage of iTNFα+ CD14+ cells compared with HDs. After 12 injections of adalimumab treatment, the percentage of iTNFα+ CD14+ cells increased progressively, reaching levels similar to those of HDs. This effective adalimumab-induced iTNFα+ CD14+ upregulation was observed only in RA patients who showed a EULAR-defined response. After the first two injections, TNFα on the membrane and in the culture supernatant was effectively blocked by adalimumab in all but one patient, who developed antiadalimumab antibodies.

TNFα plays a key role in RA physiopathology. However, we did not expect patients with active RA to have a lower percentage of iTNFα+ CD14+ cells than HDs after TLR cultures. This finding suggests that monocytes from patients with active RA have different regulation of iTNFα production. To understand this unexpected finding, we cultured HD cells with exogenous TNFα, which confirmed that TNFα signals induced negative feedback on iTNFα production or increased traffic of iTNFα to the membrane. Other authors have previously observed that TNFα induced tristetrapolin production, which destabilized TNFα mRNA [23]. Our observations therefore suggest that RA cells were exposed to TNFα. However, TNFα levels were undetectable in serum from these RA patients. We propose that alternative sources of TNFα for circulating RA cells could be local soluble or membrane TNFα [5,24]. On the basis of the results of our present experiments, we cannot exclude the potential that there are additional factors involved in the reduction of iTNFα+ CD14+ cells.

We observed that ESR and DAS28-ESR were inversely correlated with the number of iTNFα+ CD14+ cells, suggesting that iTNFα+ CD14+ cells and inflammatory activity were robustly associated. We think that the downregulation of iTNFα+ CD14+ cells percentage was a regulatory mechanism to control excessive TNFα and its detrimental consequences.

Adalimumab is currently indicated to reduce signs and symptoms in adult patients with moderately to severely active RA. It induces clinical response, inhibits the progression of joint damage and improves physical function [25]. Here we show that levels of iTNFα+ CD14+ cells increased progressively after the first two injections of adalimumab. This effect was not observed in RA patients in remission induced by methotrexate, suggesting that upregulation of iTNFα+ CD14+ cells was adalimumab-dependent. In addition, the highest levels of iTNFα+ CD14+ cells after 12 injections coincided with the greatest clinical improvement (measured by EULAR-defined response), suggesting that proper regulation of iTNFα production in RA patients was associated with a good to moderate EULAR-defined response .

In our *in vitro* experiments, adalimumab was not able to restore normal iTNFα+ CD14+ levels overnight when cells had previously been exposed to TNFα, suggesting that, *in vivo*, normalization of iTNFα+ CD14+ cells was due to indirect mechanisms. One possible mechanism is that adalimumab blocks TNFα binding to its receptor [12]. Without TNFα signaling [23], interleukin 1 (IL-1), IL-6, IL-8, granulocyte-macrophage colony-stimulating factor [10] and acute-phase reactants of inflammation (CRP, fibrinogen and ESR) [14] are reduced. This reduction implicates the return of RA cells to a basal state similar to that of cells of HDs and the consequent correction of the percentage of iTNFα+ CD14+ cells. Another possible mechanism is that the renovated circulating pool of CD14+ cells has no contact with TNFα, because TNFα is blocked [26]. A third possible mechanism is also worthy of mention. Monocytes and macrophages from RA patients are targets of TNFα signals and the major producing cells of TNFα, which they present in significant amounts in membrane [27]. Adalimumab has cytotoxic effects on mTNFα-expressing cells [28]. However, it takes several days to get rid of all the cells with mTNFα. Once they are all eliminated, new CD14+ cells with regular iTNFα production repopulate the system.

We observed that infliximab, another neutralizing anti-TNFα monoclonal antibody, restored the percentages of iTNFα+ CD14+ cells to normal values after a long period of therapy. However, in a previous study with infliximab, researchers showed that monocytes produced lower amounts of iTNFα after 24 weeks of treatment than before treatment [29]. This discrepancy in results could be due to clinical and methodological differences. The most crucial difference is that the results described in the previous report were expressed as molecules of equivalent soluble fluorescein per cell, whereas ours are expressed as the percentage of CD14+ monocytes with iTNFα. In addition, and in contrast to those authors, we also evaluated iTNFα levels in HDs to establish the normal percentages of iTNFα+ CD14 + -producing cells.

It should be noted here that, although infliximab and adalimumab are both TNFα-blocking antibodies, they are not fully comparable. Infliximab is administered intravenously at 0, 2 and 6 weeks, followed by a maintenance infusion dose every 8 weeks, whereas adalimumab is administered subcutaneously every 15 days. Moreover, infliximab is a chimeric antibody, whereas adalimumab is a fully humanized antibody.

Adalimumab treatments significantly reduced levels of free mTNFα on CD14+ cells and free soluble TNFα in TLR cultures compared to the first injection. This dramatic reduction in mTNFα on cells was due to two activities of adalimumab: (1) its binding blocked TNF signals and (2) it competed with antibodies for flow cytometry detection [17]. A similar conclusion could be reached for soluble TNFα [30]. Unfortunately, our experimental approach does not allow us to establish the fate of mTNFα and soluble TNFα after adalimumab. However, because iTNFα is the source of mTNFα and soluble TNFα, we can speculate that iTNFα, mTNFα and soluble TNFα have comparable kinetics.

Not all patients respond to adalimumab therapy [16]. In our study, patient 11 did not respond, and, consequently, no iTNFα regulation in CD14+ cells was observed. The lack of response in this patient was due to the presence of antiadalimumab antibodies after the second injection. Although patient 7 responded to adalimumab and iTNFα+ CD14+ cells increased accordingly after two injections, treatment was discontinued after the third injection due to allergy. Consequently, the percentage of iTNFα+ CD14+ cells dropped to levels seen before adalimumab therapy was started. We think that, if adalimumab had not been withdrawn, this patient could have had a good or moderate EULAR-defined response.

We hope that our ongoing experiments will allow us to determine whether the increase in iTNFα+ CD14+ cells is RA-specific or could be extended to other inflammatory pathologies in which TNFα plays a crucial role. Studying the regulation of iTNFα+ CD14+ cells in RA patients could provide individualized information about the efficacy of adalimumab treatment.

## Conclusions

Our findings suggest that adalimumab treatment in RA patients can return iTNFα levels to those of HDs. This effect was not observed in the presence of neutralizing antiadalimumab antibodies that produced no EULAR-defined response. Our future studies of iTNFα in monocytes during treatment with adalimumab therapy in RA patients could provide information about the effectiveness of treatment.

## Abbreviations

ACPA: Anticitrullinated peptide antibody; CRP: C-reactive protein; DMARD: Disease-modifying antirheumatic drug; ESR: Erythrocyte sedimentation rate; HD: Healthy donor; iGMFI: Integrated geometric mean fluorescence intensity; iTNFα: Intracellular tumor necrosis factor α; LPS: Lipopolysaccharide; LTA: Lipoteichoic acid; mTNFα: Membrane tumor necrosis factor α; RA: Rheumatoid arthritis, RF, rheumatoid factor; TACE: Metalloproteinase tumor necrosis factor α–converting enzyme; TLR: Toll-like receptor; TNFα: Tumor necrosis factor α.

## Competing interests

The authors declare that they have no competing interests.

## Authors’ contributions

CZ carried out cell culture and flow cytometry, analyzed and interpreted the experimental and clinical data, participated in the design of the study and drafted and edited the manuscript. CDT collected samples and clinical parameters, analyzed and interpreted the experimental and clinical data and participated in the design of the study. CG, CDL, JC, JLL and PM collected samples and clinical parameters. MAO carried out ELISAs and analysis of data. IC collected samples and clinical parameters and revised several sections of the manuscript. JCN carried out ELISAs and participated in the analysis of flow cytometry data. EC assisted in the cell culture design and analyzed the experimental data. CJ participated in the analysis of experimental data and revised several sections of the manuscript. SV conceived of the study, participated in the design and coordination of the study, analyzed clinical and experimental data and participated in the drafting and editing of the manuscript. All authors read and approved the final manuscript.
